# Influence of Eugenol and Its Novel Methacrylated Derivative on the Polymerization Degree of Resin-Based Composites

**DOI:** 10.3390/polym15051124

**Published:** 2023-02-23

**Authors:** Ali Alrahlah, Abdel-Basit Al-Odayni, Waseem Sharaf Saeed, Naaser A. Y. Abduh, Rawaiz Khan, Abdulrahman Alshabib, Faisal Fahad N. Almajhdi, Riad M. Alodeni, Merry Angelyn Tan De Vera

**Affiliations:** 1 Restorative Dental Sciences Department, College of Dentistry, King Saud University, Riyadh 11545, Saudi Arabia; 2 Engineer Abdullah Bugshan Research Chair for Dental and Oral Rehabilitation, College of Dentistry, King Saud University, Riyadh 11545, Saudi Arabia; 3 Department of Chemistry, College of Science, King Saud University, Riyadh 11451, Saudi Arabia; 4 Division of Biochemistry, Chemistry Department, Faculty of Science, Ibb University, Ibb P. O. Box 70270, Yemen; 5 Research Center, College of Dentistry, King Saud University, Riyadh 11451, Saudi Arabia

**Keywords:** eugenol derivative, methacrylate derivative, free radical inhibition, degree of conversion, dental composites

## Abstract

The aim of this work was to assess the limiting rate of eugenol (Eg) and eugenyl-glycidyl methacrylate (EgGMA) at which the ideal degree of conversion (DC) of resin composites is achieved. For this, two series of experimental composites, containing, besides reinforcing silica and a photo-initiator system, either EgGMA or Eg molecules at 0–6.8 wt% per resin matrix, principally consisting of urethane dimethacrylate (50 wt% per composite), were prepared and denoted as UGx and UEx, where x refers to the EgGMA or Eg wt% in the composite, respectively. Disc-shaped specimens (5 × 1 mm) were fabricated, photocured for 60 s, and analyzed for their Fourier transform infrared spectra before and after curing. The results revealed concentration-dependent DC, increased from 56.70% (control; UG0 = UE0) to 63.87% and 65.06% for UG3.4 and UE0.4, respectively, then dramatically decreased with the concentration increase. The insufficiency in DC due to EgGMA and Eg incorporation, i.e., DC below the suggested clinical limit (>55%), was observed beyond UG3.4 and UE0.8. The mechanism behind such inhibition is still not fully determined; however, radicals generated by Eg may drive its free radical polymerization inhibitory activity, while the steric hindrance and reactivity of EgGMA express its traced effect at high percentages. Therefore, while Eg is a severe inhibitor for radical polymerization, EgGMA is safer and can be used to benefit resin-based composites when used at a low percentage per resin.

## 1. Introduction

Eugenol (4-allyl-2-methoxyphenol) (Eg) is the major volatile, biologically active compound of clove oil. It is a versatile chemical classed as a phenylpropanoid of the allyl-phenol type and has a lengthy history of use in dentistry [1]. In this regard, Eg is best known for its original use as a cavity-filling temporary cement, typically in a zinc oxide-Eg paste. It possesses analgesic and anti-inflammatory properties to relieve irritated or diseased tooth pulp pain. As a consequence of its desirable properties, it has also been implicated in different industries, including food, cosmetics, medicines, and pharmaceuticals [1,2,3]. It is a powerful antioxidant with ready radical-scavenging properties, making it a potent free radical inhibitor. Owing to its negative effect on radical polymerization of restorative composites [4], Eg incorporation within permanent filling materials is therefore unfavored.

On the other hand, modification of Eg’s structure with the polymerizable (meth)acrylate group could be beneficial, preserving the structural advantages of the Eg moiety rather than the negative inhibitory impact on radical polymerization, thus allowing its participation in resin-based restorative dental composites. Indeed, the Eg molecule is chemically sensitive under certain conditions and, when exposed to light, for example, it may degrade into radicals such as hydrogen, phenoxyl, and allyl, which are responsible for its antioxidation and free radical polymerization inhibition effect. However, the activity of allyl radicals is known to be poor. This implies that the modification of Eg’s structure with (meth)acrylate-based functional groups through phenoxyl-oxygen is preferred for various applications involving radical polymerization.

Dental resin composite is comprised of several components [5]: (i) organic matrix, which is a mix of monomers, typically of acrylate type, which form the resin network upon polymerization; (ii) reinforcing material, that is commonly of silica type and some other oxides; (iii) a coupling agent, which consists of organosilane and serves as an organic–inorganic interface linkage; (iv) an initiating system, which comprises a photo-initiator, co-initiator, and radical accelerator, and (v) additives, materials used for specific purposes, such as pigments, antibacterial agents, etc. Hence, monomers are typically used as a base for other components and are liable for hardening the composite. They are the main component of the organic phase or matrix and, when exposed to light, they easily polymerize. Matrices of conventional dental composites commonly comprise one or more of the (meth)acrylate-based monomers, usually of free radical multi-polymerizable functionalities, such as bisphenol A-glycidyl methacrylate, urethane dimethacrylate (UDMA), and triethylene glycol dimethacrylate [6]. However, incorporation of other (meth)acrylates, including mono-functional monomers, for beneficial purposes within the matrix could also be an advantage [7].

Recently, Eg-derived (meth)acrylates have attracted researchers’ attention, and the proceeding efforts are tangible. For example, eugenyl methacrylate and ethoxyeugenyl methacrylate [8], ethoxy eugenyl acrylate [9], and eugenyl-2-hydroxypropyl methacrylate (EgGMA) [10] were synthesized through etherification or esterification of the eugenol-phenoxy group; however, other derivatives fabricated via the allyl terminal were also reported [9,11,12]. Most of these polymerizable Eg derivatives were evaluated for their physicochemical, biocompatibility, and applicability as additives to resin-based dental composites with the potential to act as bactericidal and physicochemical enhancers [7,13,14].

EgGMA, a new, recently introduced monomer, has been investigated for its monomeric chemical, thermal, and reactivity properties [10], as well as for its potential incorporation in resin-based dental composites [7,14], reporting comparable features to that of MMA with better biocompatibility and water sorption against EgGMA-free composites. However, some properties, such as the degree of conversion (DC) and water solubility, were slightly affected, thus necessitating further investigation. The DC, in particular, is a crucial determiner of majority of the final composite properties; thus, according to a previous study [14] in which DC was evaluated for 0–17 mol% per organic phase, it is an EgGMA concentration-dependent property. However, the analyzed concentrations were limited to 0.00, 5.45, and 16.66 mol% and, therefore, a wider analysis with a small concentration increment is important to find the valuable upper concentration of EgGMA in the matrix.

In this work, the objective was to determine the maximum concentration of EgGMA that would benefit the resin composite without sacrificing the DC, and to compare this with the radical inhibitory effect of its Eg precursor. It is hypothesized that: (1) the addition of Eg and EgGMA will not significantly affect the DC of the prepared model composite, (2) the DC would not significantly differ with Eg or EgGMA concentration increases from 0 to 10 mol% per matrix, and (3) there is no significant differences in the DC of Eg- and EgGMA-containing composites at the same mole fraction per matrix.

## 2. Materials and Methods

### 2.1. General Chemistry 

UDMA (>97%), Eg (98.5%), glycidyl methacrylate (GMA, 98%), CQ (97%), 2-(dimethylamino)ethyl methacrylate (DMAEMA, 98%), 3-(trimethoxysilyl)propyl methacrylate (γ-MPS, 98%), hydroquinone (HQ, >99%), ethanol absolute (EtOH, ≥99.8%), and tetraethyl orthosilicate (TEOS, 98%) were procured from Sigma–Aldrich (Taufkirchen, Germany). Triphenylphosphine (Ph_3_P, 99%) was purchased from Cica-reagent Kanto Chemical Co. (Tokyo, Japan). Ethyl acetate (EA, 99.5%), *n*-hexane (*n*-H, 95%), and ammonium hydroxide (NH_4_OH, 35%) were obtained from Fisher Scientific (Loughborough, UK).

The EgGMA monomer was synthesized through a mediated ring-opening route of GMA-epoxy combined with catalytic etherification of Eg, as previously described [10]. Typically, in a three-necked round-bottom flask, equimolar concentrations of reactants Eg and GMA were first homogenized with stirring, then 0.5 wt% (with respect to reactants’ total mass) each of the inhibitor HQ and the catalyst Ph_3_P was added, and the reaction was maintained under nitrogen atmosphere at 120 °C, with a reflex for 2 h. The reaction progression was assessed using thin-layer chromatography (TLC; an aluminum plate of a silica-based 0.5 mm layer stationary phase and 3:7 EA/*n*-H mobile phase). The product EgGMA was purified using silica gel column chromatography using the above-mentioned mobile phase as an eluent.

Silanized silica with a target particle size of 500 nm was prepared using the Stöber method [15]. Briefly, water (40 mL), EtOH (250 mL), and ammonium hydroxide (25 mL) were mixed, cooled in an ice bath before initial addition (dropwise during 5 min) of TEOS (45 mL), and then brought to room temperature. Next, a second portion of TEOS (33 mL) in EtOH (250 mL) was added, and the mixture was left to react for 8 h. Subsequently, γ-MPS (10 vol% with respect to TEOS) was added, and the reaction mixture was stirred overnight. The obtained silanized silica was collected by centrifugation, washed 3 times with EtOH using a suspension–centrifugation process, then the solvent was removed, and particles were dried under a vacuum overnight.

The FTIR spectroscope, a Nicolet iS10 FTIR spectrometer from Thermo Scientific (Madison, WI, USA), equipped with an attenuated total reflection (ATR, diamond crystal) chamber, was used for material and DC analysis. Each ATR-FTIR spectrum was obtained at a 4 cm^−1^ measuring resolution and as an average of 32 cycles at room temperature. The particle size, dispersity, and morphology of the synthesized silanized silica were confirmed using a scanning electron microscope (SEM) (JEOL, JSM-6610 LV, Tokyo, Japan) at 15 kV voltage and a resolution of ×10,000.

### 2.2. Preparation of Experimental Composites

**Figure 1:**
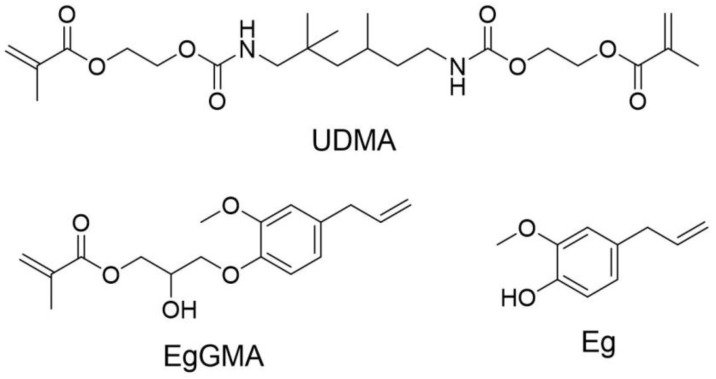
Chemical structures of urethane dimethacrylate (UDMA), eugenyl glycidyl methacrylate (EgGMA), and eugenol (Eg).

Two series of resin-based dental composites containing a 50 wt% resin matrix (UDMA with either EgGMA or Eg, Figure 1) and 50 wt% silanized silica were prepared as presented in Table 1, and each composite contained 0.5 wt% of both the initiator (CQ) and co-initiator (DMAEMA). EgGMA or Eg in the matrix were predesigned to be in the range of 0–6.8 wt% (EgGMA = 0–10 mol%). In a typical procedure, composites with the highest (UG6.8 or UE6.8) and the lowest (UG0 = UE0 control, a diluent) concentrations of either EgGMA or Eg were separately prepared, in excess. Thus, the initiator components were dissolved in the target resin, followed by filler addition. The composite was manually mixed using a stainless-steel spatula, then further homogenized in a speed mixer (TM DAC 150 FVZ, Hauschild and Co., Hamm, Germany) three times (for 1 min each, with 2 min rest in between) at 3000 rpm. Then, a series of UG and UE composites were obtained by mixing the appropriate percentage of these two composites and, to ensure homogeneous composition, composites were again manually and mechanically mixed as above. Composites were kept cooled in dark containers until use.

### 2.3. Measurement of Degree of Polymerization

To ensure similar conditions during measurements, specimens were prepared in a fabricated disk-shaped, stainless-steel mold of 5 × 1 mm. The pastes were compacted in the mold, and their FTIR spectra were recorded (uncured samples). Further, both sides of the specimen-containing mold were covered with layers of plastic strips and glass slides and irradiated for 60 s from one side using an LED light-curing unit (Bluephase, Ivoclar Vivadent, Schaan, Lichtenstein), with 650 mW/cm^2^, a wavelength range of 385 to 515 nm, and a 10 mm rotatable tip. The light intensity was monitored using an Optilux Radiometer (Kerr Corp., Danbury, CT, USA), and the tip was set in direct contact with the specimen surface. After 10 min of polymerization, the ATR-FTIR spectra were recorded (cured samples) using the preset FTIR condition. Five replicates (*n* = 5) were prepared for each composite, and each specimen disk was measured (cured side) at least three times, placed at the center and at 1 to 2 mm from the center, and the calculated DCs were averaged.

The DC was calculated based on the change in the stretching peak of the vinylic double bond (C=C) at 1637 cm^−1^ compared to the band of C-H bending mode at 1453 cm^−1^ as an internal standard peak [16]. Thus, the areas of the target peaks were procured from the corresponding spectra, and DC was calculated using Equation (1):

(1) $$\mathrm{DC}\;\left(\%\right)=\left[1-\frac{{\left(\frac{A_{1637}}{A_{1453}}\right)}_{\mathrm{cured}}}{{\left(\frac{A_{1637}}{A_{1453}}\right)}_{\mathrm{uncured}}}\right]\times 100$$

where A_1637_ and A_1453_ were the areas (mole fractions) under the peaks at 1637 and 1453 cm^−1^ for aliphatic C=C stretching and C-H bending modes in the same spectra, before and after light-curing.

### 2.4. Statistical Analysis

Data for DCs from the two groups (UG and UE) were statically analyzed using SPSS ver. 21 (IBM Corp., Armonk, NY, USA). One-way ANOVA followed by Tukey post-hoc and Bonferroni multiple comparison tests were used to analyze the significant differences between the two groups at a *p*-value < 0.05.

## 3. Results

### 3.1. Material Characterization

**Figure 2:**
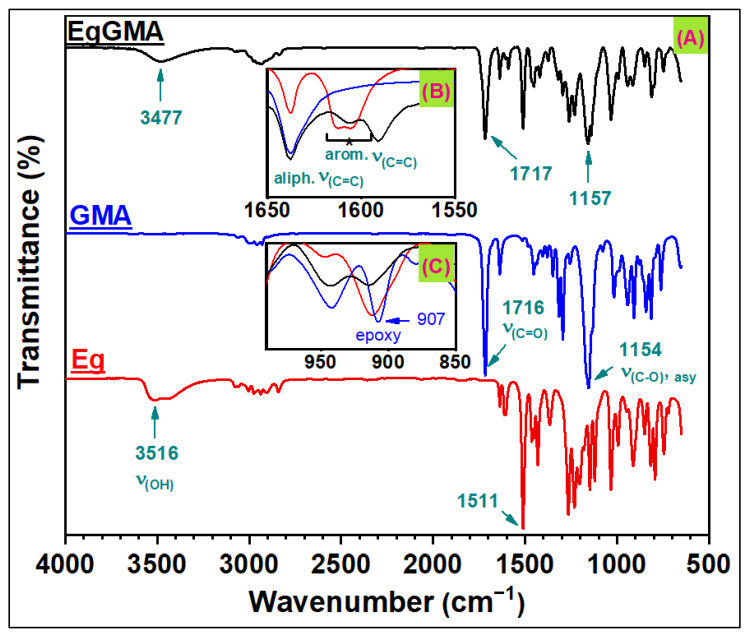
(**A**) Fourier transform infrared (FTIR) spectra of eugenol (Eg), glycidyl methacrylate (GMA), and eugenyl-glycidyl methacrylate (EgGMA). Inserts: (**B**) the spectra range between 1650 and 1550 cm^−1^ of aliphatic and aromatic C=C bonds, and (**C**) the absorption range of the epoxy ring.

The success of EgGMA synthesis was confirmed by FTIR analysis. Figure 2 shows the FTIR spectra of EgGMA and its precursors Eg and GMA. The spectrum of EgGMA differed from that of Eg and GMA. For example, Eg phenolic hydroxyl’s absorption profile comprised two peaks that were for free and hydrogen-bonding OH at 3516 and 3451 cm^−1^, respectively, whereas the EgGMA secondary OH revealed only one peak centered at 3477 cm^−1^. Moreover, the possible ring resonance of Eg could be proven by the two overlapped peaks centered at 1612 and 1606 cm^−1^ assigned to aromatic C=C of various aromatic structures, of which Eg and quinomethide were the most discerned. However, such an absorption profile was not seen in the EgGMA spectra due to infeasible quinone generation, thus confirming the success of the etherification reaction (Figure 2B, inset B) [17]. Furthermore, the peak characteristic of the epoxy ring in GMA at 907 cm^−1^ was shifted to 915 cm^−1^ in EgGMA, indicating ring-opening (Figure 2C, inset C) [18,19,20]. The structural integrity of EgGMA was confirmed by monitoring the peak of C=C aliphatic bonds around 1606 cm^−1^ for vinyl and allyl functionalities, as seen in Figure 2.

**Figure 3:**
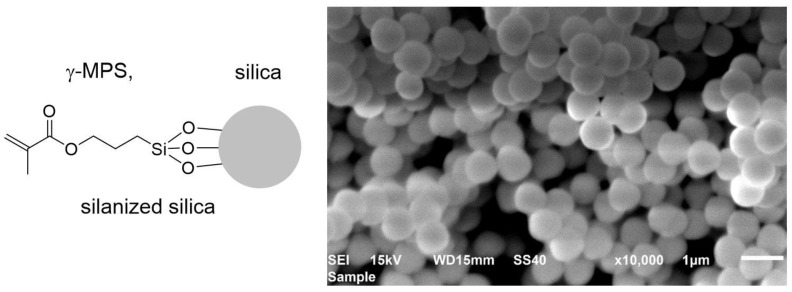
Scanning electron microscope (SEM) image of the synthesized silanized silica. The image scale bar is 1 µm.

Figure 3 shows the SEM image of the synthesized silanized silica. The micrograph displays spherical, low-dispersity particle sizes between 500 and 700 nm.

### 3.2. Double-Bond Conversion

**Figure 4:**
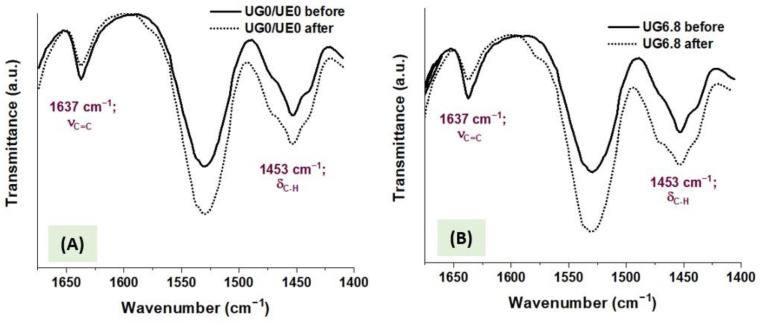
FTIR peaks in the range between 1675 and 1410 cm^−1^ of selected composites: the control (UG0 = UE0) (**A**), and composites with the highest rate of EgGMA (UG6.8) (**B**), before and after the light-curing process.

**Figure 5:**
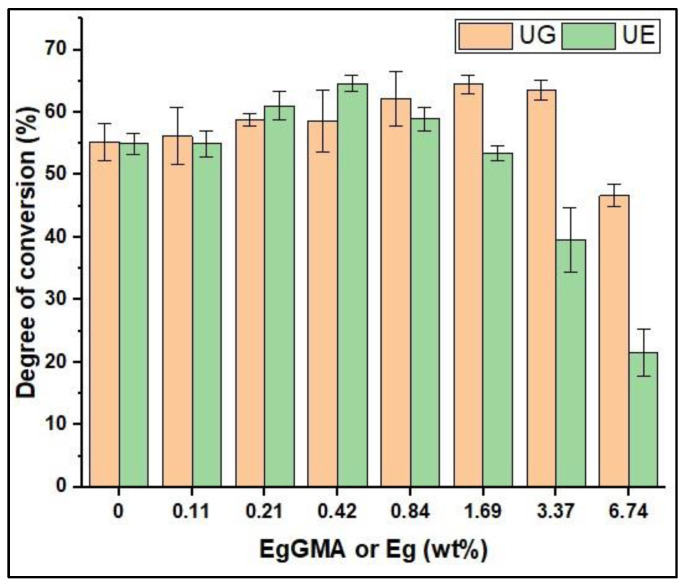
Degree of conversion (DC) of investigated composites: UG0–UG6.8 and UE0–UE6.8 groups. Bars are for standard deviations of five replicates (*n* = 5) per composite. UG is urethan dimethacrylate (UDMA)-eugenyl glycidyl methacrylate (EgGMA), UE is UDMA-eugenol (Eg), and numbers indicate the weight percent fraction of EgGMA or Eg (0–6.8 wt%) in the matrix.

Figure 4 illustrates the ATR-FTIR spectra of selected composites: those of EgGMA-free composites (UG0) and those of composites with the highest examined EgGMA concentration (UG6.8) before and after light-curing. In these spectra, the peaks at 1637 and 1453 cm^−1^ represent the stretching mode of aliphatic C=C and the bending vibration of C-H bonds, in which the intensities (peak areas) elucidate the residual mole fraction of vinyl groups in reference to C-H as an internal standard, before and after polymerization. The computed data from the FTIR spectra were utilized for the DC calculation, and the average of five replicates for each composite are gathered in Table 2 and depicted, with standard deviation bars, in Figure 5.

According to the data, the overall impact of EgGMA and Eg on composite DC is concentration-dependent, and effective DC is material-dependent; thus, the first null hypothesis stating that there would be no effect of incorporating Eg or EgGMA on the DC of the model composite is rejected, and the second hypothesis stating that Eg and EgGMA concentrations have no significant effect on the DC of the composite was partially rejected as the effect become significant only after a certain percentage, as discussed hereafter. It was noticed that the highest DCs were 65.42% and 65.06% at UG1.7 and UE0.4, respectively, while the negative effects were suggested above UG3.4 and UE0.8. The DCs of UG6.8 and UE6.8 at the highest tested wt% were 47.08% and 21.54%, respectively. It is worth mentioning that in the same column in Table 2, the UG wt% was the same as UE, while its mol% was almost the half the UE; thus, mole percentage may also be compared to show how significantly the inhibitory effect of Eg is reduced after modification.

As can be seen, no statistically significant differences (*p* > 0.05) in DC between UG- and UE-based composites at low concentrations up to 0.8 wt% were detected, beyond which the DCs significantly differentiated; therefore, the third hypothesis is partially rejected as the significant differences have been observed only above a certain concentration, i.e., 2.5 mol%. Furthermore, a detrimental drop in DC from 63.87% to 47.08% in the case of EgGMA-incorporating composites UG3.4 and UG6.8 was noticed, suggesting a EgGMA incorporation limit of 3.4 wt% (5.13 mol%) per matrix. On the other hand, the Eg addition limit was greatly lower, with severe inhibition at a fraction higher than 0.8 (2.17 mol%) per matrix (Table 2).

## 4. Discussion

This study was designed to compare the effect of Eg and its methacrylated derivative (EgGMA) on the free radical polymerization of resin-based composites, by employing a range of initial fractions of 0–6.8 wt% per matrix of either Eg or EgGMA. The comparison was performed based on a transformation of the polymerizable vinylic double bond (C=C) into a single bond (C-C) upon photo-irradiation, measured using the ATR-FTIR technique. The experimental composites were formulated as presented in Table 1, in which the 50 wt% resins combined UDMA with either Eg or EgGMA monomers. The DC was calculated using the FTIR method based on Equation (1).

### 4.1. Degree of Conversion (DC)

The value of DC of dental composites is associated with several factors, including resin type and concentration, fillers, and curing conditions, and therefore, standardization is difficult. From a practical perspective [21], no minimum value for a clinically satisfactory restoration has been precisely established yet; however, a DC higher than 55% was suggested as acceptable. As seen in Figure 5, the DC of UG composites was gradually increased as the EgGMA concentration increased up to 3.4 wt% (5.13 mol%) per resin phase, then decreased when EgGMA increased up to 6.8 wt% (UG6.8). According to the statistical analysis, the DC increase became significant above 0.8 wt% (1.3 mol%) compared to the control (UG0) (*p* < 0.05). The possible cause of such enhancement in DC is the viscosity differences between UDMA and EgGMA, which seemingly reduced the overall viscoelasticity of the composites. However, the observed drop in the DC of UG6.8 may be due to both the monomers’ structure and reactivity differences [10]. This could support the potential use of EgGMA in dental composites at a low but effective concentration, as previously reported [7,14].

In contrast, the DC of composites containing the Eg molecule revealed an earlier inhibitory effect as its concentration reached 0.2 wt% (UE0.2, 0.54 mol%). Such increase in DCs of UE0–UE0.4–UE0.8 may reflect the viscosity effect rather than inhibition, and this case was evinced by a significantly instant increase (*p* < 0.05) in DC from 56.70% for the control to 61.34% for UE0.2, which continued increasing up to UE0.4, after which DC started to gradually decline down to UE1.7 (54.99%). However, by further increasing the Eg fraction, a significant drop in DC (*p* < 0.05) was observed down to 21.54% for UE6.8, indicating severe inhibition of radical polymerization.

DC, however, represents the degree to which monomers have been converted into polymers. Therefore, an incomplete curing of the composite will result in higher residual monomers after curing. The uncured monomers are prone to being released from the composites, compromising the restoration and promoting secondary decay and infections [22]. As stated above, a DC below a minimum threshold of 55% is clinically insufficient, thus causing restorations to be weak and less resistant to wear [21].

### 4.2. Structure–Activity: A Comparison

**Figure 6:**
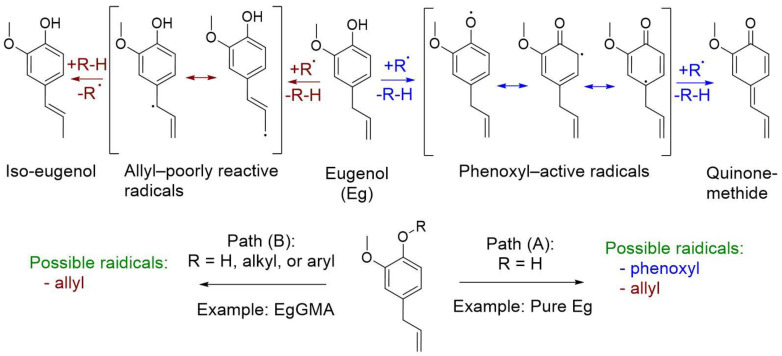
Phenoxyl and allyl radicals of eugenol and eugenol derivatives.

Eugenol (Figure 1), 4-allyl-2-methoxyphenol, is an allylic substituted guaiacol (2-methoxyphenol) compound that, under certain circumstances, is well-known to possess prooxidant and antioxidant activities [23,24]. The inhibitory action of Eg on radical polymerization is associated with the production of phenoxyl radical species (Figure 6) due to its ready scavenging activity. The generating radicals may cause inhibition or retardation of the polymerization reaction depending on the concentration of existing radicals in the mixture. Such characteristic is defined by the induction period; that is, the true inhibitor is consumed first, then polymerization occurs as normal, while retarders lead to a temporary reduction of the reaction rate due to their slow degradation to radicals. The inhibition effect of Eg at a high concentration may be due to its ready radical-scavenging properties, which cause consumption of initiator-based radicals necessary for effective polymerization [4,24,25,26].

Additionally, the allyl double bond is less reactive than the vinyl one, and its radicals are much more stable. Propagation is quickly self-terminated by hydrogen abstraction from an allylic monomer in a so-called degradative chain transfer mechanism [10], leading to resonance-stabilized radicals. Even though this seems an acceptable assumption, and to the best of our knowledge, no ploy(eugenol) synthesized by free radical polymerization has been reported, it appears that large quantities of initiator free radicals are required which, in turn, results in short chains and large amounts of terminal initiator end groups that are residuals of initiator decomposition products and may function as effective impurities as well.

By monitoring the DC of the investigated composites, via the Eg fraction increase, DC gradually increased up to 0.4 (1.09 mol%), i.e., from 55.10% of Eg-free composite (the control) to 65.06% of UE0.4. However, by further increasing the Eg fraction, DC began to decline, with no significant differences compared to the control up to UE1.7 (DC = 54.99%) (*p* > 0.05), above which a noticeable drop to DCs of 40.97% and 21.54% for UE3.4 and UE6.8, respectively, was observed, representing significant differences (*p* < 0.05) at the defined ratio. This behavior may suggest a viscosity effect which slightly increases with Eg addition (Eg is less viscous than UDMA). Due to the effect of Eg-based radicals at a high concentration, which may cause depletion of initiator radicals and quenching of chain growth, the observed increase in DC was not a straightforward case [9,27]. Despite the numerous studies on the inhibitory effect of Eg, the mechanism behind the inhibition still not well-understood [28,29].

On the other hand, the chemical structure of EgGMA comprises the Eg moiety, isopropanol structure, and methacrylate-based active terminal, as shown in Figure 1. Compared with the Eg structure, no phenoxyl radicals on the EgGMA molecule can be generated, suggesting the absence of radical-scavenging activities through this degradative mechanism (Figure 6), and thus polymerization inhibition due to such activity is not expected. As seen above for UGs, the degree of polymerization was increased as the EgGMA mole fraction increased up to 1.7 wt% (2.67 mol%), reaching 65.42%, which is higher than the control by 11.02% but close to that of UE at a higher DC (UE0.4, 65.06%). As DC increased and the difference between UG and UE composites at a low concentration was not statically significant (*p* > 0.05), the inhibitory impact was seemingly negligible, but the viscosity was the dominant influencer. Even though the viscosity of EgGMA and Eg was not analyzed in this work, their magnitudes were assumed to be in the order of UDMA >>> EgGMA > Eg, and thus UDMA dilution by Eg could be more effective than EgGMA, supporting the DC difference observed when Eg and EgGMA were incorporated. However, other factors can affect the DC of the composites, as discussed above for Eg-containing ones. In the case of UGs, the decline in DC at a high EgGMA concentration (i.e., for UG6.8) could be assigned to the reactivity and steric hindrance effects of the EgGMA monomer compared to UDMA [10,30]. According to the literature [9,10], the reactivity of Eg-derived (meth)acrylates is affected by the presence and reactivity of the allyl group, which can also be involved in radical addition (cross-propagation), degradative chain transfer, and the termination reaction. Moreover, a study resulting in 58% conversion of the methacrylate double bond reported only 6% of allyl converted into a single bond, simultaneously with the vinyl group in the same Eg-derived methacrylate monomer [9]. Meanwhile, allyl’s contribution in this manner is more likely to produce a branched structure network, and its participation in post-polymerization and cross-linking network formation is possible as well [2,31].

## 5. Conclusions

The DC of resin composites containing either Eg or EgGMA monomers incorporated at 0–6.8 wt% with respect to the organic phase of the composite, which in turn comprised 50 wt% matrix and 50 wt% modified silica, was studied with the aim to match the concentration that exerts an inhibitory effect on the radical polymerization. EgGMA and Eg have enhanced polymerization at concentrations of less than 6.8 and 1.7 wt%, respectively, with a proven vanishing inhibitory effect common to the Eg moiety. Furthermore, a slight, but statically significant, reduction in the DC at 6.8 wt% of EgGMA was observed, which was assigned to the reactivity differences between UDMA and EgGMA and the steric hindrance caused by the bulky EgGMA residual structure. Consequently, the findings support the possible incorporation of EgGMA within the dental resin matrix at 5 mol% or less, encouraging its further investigation as a permanent bioactive material within restorative resin composites.

## Figures and Tables

**Table 1:** Composition of the investigated experimental composites, showing the variation of EgGMA and Eg in the matrix of UDMA.

Matrix Composition of UG Series					Matrix Composition of UE Series				
Code	UDMA		EgGMA		Code	UDMA		Eg	
	mol%	wt%	mol%	wt%		mol%	wt%	mol%	wt%
UG0	100	100	0.00	0.00	UE0	100	100	0.00	0.00
UG0.1	99.84	99.90	0.16	0.11	UE0.1	99.73	99.89	0.27	0.11
UG0.2	99.68	99.79	0.32	0.21	UE0.2	99.46	99.79	0.54	0.21
UG0.4	99.36	99.58	0.64	0.42	UE0.4	98.91	99.58	1.09	0.42
UG0.8	98.72	99.16	1.28	0.84	UE0.8	97.83	99.15	2.17	0.85
UG1.7	97.33	98.32	2.67	1.69	UE1.7	95.66	98.3	4.34	1.70
UG3.4	94.87	96.63	5.13	3.37	UE3.4	91.32	96.61	8.68	3.39
UG6.8	89.74	93.26	10.26	6.74	UE6.8	82.64	93.22	17.36	6.78

In the two series, each composite consists of 50 wt% resin and 50 wt% silanized silica. The initiator camphorquinone (CQ) and the co-initiator 2-(dimethylamino)ethyl methacrylate (DMAEMA) were both added as 0.5 wt% per resin mass.

**Table 2:** Degree of conversion (DC) of the experimental composites, UG and UE groups (*n* = 5).

Composite	Degree of Conversion (%); Standard Deviation in Parentheses						
	UG0 or UE0	UG0.2 or UE0.1	UG0.4 or UE0.2	UG0.8 or UE0.4	UG1.7 or UE0.8	UG3.4 or UE1.7	UG6.8 or UE3.4
Approximate mole% of Eg = EgGMA per matrix	0.0 mol%	0.3 mol%	0.6 mol%	1.3 mol%	2.5 mol%	5 mol%	10 mol%
UG group	55.10 (1.80)	59.86 A (2.78)	60.23 A (4.21)	63.22 A,* (3.16)	65.42 A,* (1.49)	63.87 A,* (1.78)	47.08 A,* (1.32)
UE group		56.46 A (2.45)	61.34 A,* (1.69)	65.06 A,* (1.79)	59.51 B,* (1.40)	54.99 B (3.21)	40.97 B,* (4.11)

Note: Within each column, the different uppercase letters (A, B) indicate significant differences at *p* < 0.05. In the same row, the (*) is assigned for significant differences at *p* < 0.05 compared to the controls (UG0 = UE0).

## Data Availability

The data that support the findings of this study are included within the article.
